# An Implementation Evaluation of the Comprehensive Addiction Recovery Act (CARA) Policy in New Mexico

**DOI:** 10.1007/s10995-023-03787-1

**Published:** 2023-10-18

**Authors:** Nicholas Sharp, Jessi Fuchs, Amy Drake

**Affiliations:** New Mexico Department of Health, 2040 S. Pacheco, Santa Fe, NM USA

**Keywords:** CARA, Prenatal substance use, Non-punitive

## Abstract

**Purpose:**

The purpose of this field report is to describe an evaluation of the fidelity with which the comprehensive addiction and recovery act (CARA) policy has been implemented in New Mexico.

**Description:**

The CARA program in New Mexico focuses on providing nonpunitive supportive care for pregnant people affected by substance use and on coordinating services for parents, caregivers, and family members of newborns affected by substance exposure. The evaluation used information from program reports, a family follow-up survey, the plan of safe care database, and a data linkage between CARA participant records with Medicaid claims data.

**Results:**

Follow-up survey data substantiated the program reports. Both sources showed that families were not engaged consistently in developing or receiving information about plans of safe care. The survey answers also indicated that the time-period immediately after the delivery of a baby is not the best time to communicate the contents of the plan of safe care to families. Additionally, the survey found that respondents believed that medical staff judged them for using substances during pregnancy. The Medicaid data linkage showed that 40.3% of families of infants exposed to substances in-utero did not receive a plan of safe care. Program reports revealed that limited resources existed for implementing CARA.

**Conclusion:**

Program funding, limited system capacity, lack of systematic screening for prenatal substance use, regional differences in access to care, and provider biases toward pregnant people using substances affected health-care workers’ ability to identify at-risk families and develop plans of safe care. To support CARA implementation, healthcare systems must implement universal prenatal substance use screening, increase the level of anti-bias training pertaining to substance use, increase hospital supports, and improve data management systems.

**Supplementary Information:**

The online version contains supplementary material available at 10.1007/s10995-023-03787-1.

## Significance

*What is already known on this subject?* Stigma towards pregnant people for maternal substance use reduces access to prenatal care due to fear of involvement from child protective services. Punitive approaches to perinatal substance use result in lower healthcare utilization and opportunities to treat substance use disorder.

* What this study adds?* This field report evaluates the implementation of a non-punitive state level policy meant to increase services for pregnant people using substances and reduce reports to child protective services for abuse neglect due to infants exposed to substances prenatally.

**Supplementary Information:**

The online version contains supplementary material available at 10.1007/s10995-023-03787-1.

## Purpose

Perinatal substance use is a growing, significant public health issue across the nation, and strategies to identify and treat people struggling with substance use disorder vary by state (Hirai et al., 2021). Nationally, in 2020, 8.4% of pregnant people between the ages of 15 and 44 used tobacco, 10.6% used alcohol, 8.0% used marijuana, and less than 1% used opioids or cocaine in the last month (Substance Abuse & Mental Health Services Administration, 2020). Perinatal substance use is more common in New Mexico compared to the national rate. [Pregnancy Risk Assessment Monitoring System (PRAMS), 2020] In 2020, 14.3% of women in New Mexico with live births reported using marijuana/cannabis during pregnancy and 3.4% used prescription pain relievers (such as hydrocodone, oxycodone, or codeine) (PRAMS, 2020). Substance use during pregnancy, especially alcohol, tobacco, opioids, and benzodiazepines use, can cause poor infant outcomes including withdrawal symptoms, developmental delays, and birth defects (Guille & Aujla, 2019). Continued substance use after birth has been associated with an increased risk of adverse childhood experiences (Guille & Aujla, 2019). When parents continue to use substances, child protective services can become involved (Prindle et al., 2018). Furthermore, stigma that occurs in medical settings against people who use substances during pregnancy is documented as an explanation for why nearly one-third of individuals who need care do not access or engage in care even when these individuals perceive a need for it (Ashford et al., 2018).

The federal Comprehensive Addiction and Recovery Act (CARA) of 2016 was passed to address different aspects of the opioid use crisis in the United States (Congress, 2016). This policy requires all state child welfare agencies to ensure every infant born exposed to substances receives a plan of safe care, that a reporting form for infants exposed to substances in-utero is filled out, and that the number of infants receiving a plan of safe care are reported to the federal government.

New Mexico passed state legislation, House Bill 230, in 2019, also referred to as CARA,^1^ because of concerns that pregnant people were experiencing discriminatory toxicology screening and treatment under the federal CARA legislation. New Mexico created systemic changes to provide a non-stigmatizing, equitable response to perinatal substance use. The state legislation amended the Children’s Code in New Mexico to: (1) require a hospital social worker or nurse to create the plan of safe care with the family after an infant is born and before discharge, for all newborns with a substance exposure (both illicit and non-illicit); (2) send the plans of safe care to New Mexico Children, Youth and Families Department and the New Mexico Department of Health; (3) require managed care organizations (MCOs)—or Children’s Medical Services for uninsured families— to provide care coordination for families with a plan of safe care; and (4) ensure that substance use in pregnancy should not, by itself, be considered a reason for a mandatory child abuse report.

Led by the New Mexico Children, Youth and Families Department and the New Mexico Department of Health, a workgroup consisting of healthcare providers, insurance care coordinators, state agency representatives, and other stakeholders collaborated to articulate and implement New Mexico’s CARA legislation. CARA implementation involved training hospital staff, MCO care coordinators, and other service providers on the rules and regulations for CARA, which included reporting plans of safe care; providing equitable non-stigmatized care; and providing supports through care coordination. The New Mexico Children, Youth and Families Department was the implementing agency under the New Mexico CARA statute and received federal funding to support two full-time CARA positions responsible for implementing and developing a cloud-based reporting system to receive plans of safe care. The New Mexico Department of Health used other staff to support data management and implementation, but these other individuals were not dedicated positions to CARA.

An evaluation of CARA policy implementation in New Mexico was conducted to provide information about the implementation of the CARA policy and assess the impact of the CARA legislation and plans of safe care on systems and health outcomes.

## Description

### Evaluation Design Overview

**Fig. 1:**
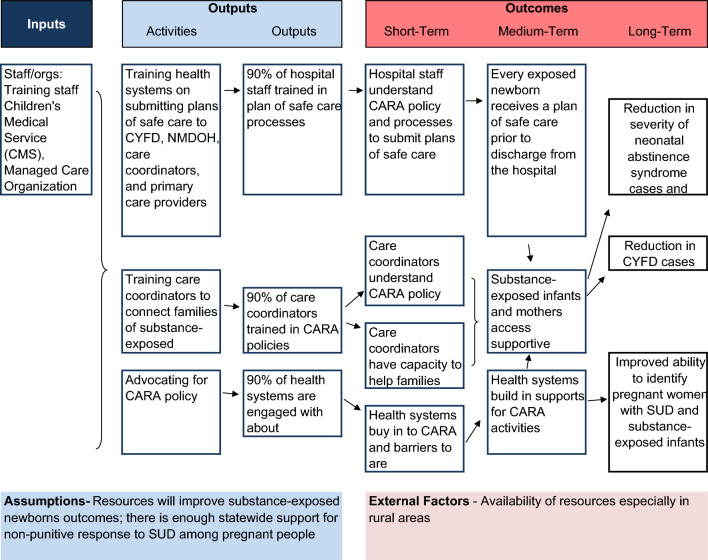
Logic model for CARA evaluation

The CARA evaluation, including the logic model (Fig. 1), evaluation questions, indicators, standards, and data sources (Table 1) were developed in coordination with the CARA workgroup described previously. This group included representatives from the New Mexico Children, Youth and Families Department, New Mexico Department of Health, Medicaid leadership, healthcare providers with expertise in treating perinatal substance use, and other community groups. The evaluation plan includes implementation and outcome evaluation phases. This paper reviews implementation and outcome data currently available.

**Table 1:** Evaluation plan overview

Phase 1			
Evaluation questions	Indicators	Standards	Data sources
Implementation evaluation			
Has the policy been implemented as intended?	Proportion of birthing hospitals in NM that have implemented protocols for plans of safe care reporting for infants exposed to substances in-utero and their families Proportion of care coordinators that have been trained in CARA rules Proportion of primary care physicians trained in plans of safe care and portal	Annually, 100% of infants exposed to substances in-utero and their families receive a plan of safe care Annually, 100% of families have plan of safe care plan of safe care explained to them and participate in developing the plan 100% of MCO care coordinators trained in CARA rules annually By 2020, 90% of primary care physicians are trained and submitting plans of safe care in portal	CARA Database/Medicaid claims database Family follow-up survey Program reports
What are barriers and facilitators to program implementation?	Barriers and facilitators associated with program planning, referral process, linkage of care, and data sharing/interoperability Feedback on provider experiences	Description of barriers and plans to address barriers	Family follow-up survey Program reports Survey of service providers
Outcome evaluation			
What benefits are infants exposed to substances in-utero and their families receiving?	Proportion of families with infants exposed to substances in-utero that are connected with a care coordinator and receive services Resources patients are referred to, follow up status, and duration of services Feedback on family experiences Patients connected with primary care physician	Annually, 90% of families with infants exposed to substances in-utero are connected with a care coordinator Annually, 90% of families with infants exposed to substances in-utero receive recommended services (include availability and reception) Description of resources patients are referred to, follow-up status, and duration of services Description of family experiences during pregnancy, and after delivery as well as perceived benefits of plan of safe care Proportion of families connected with primary care physician	CARA Database/Medicaid data Family follow-up survey FIT (Home Visiting, and Early Intervention) Children, Youth and Family Department Tracking Database/Family Infant Toddler Early Intervention Program and Children’s Medical Services databases/ CARA Database/Medicaid data tapes

### Data Sources

#### CARA Database

Plans of safe care data included demographics, substance exposures, and referred services as well as other evaluation data, including care coordination status; New Mexico Children, Youth and Families Department history; and survey data from a follow-up survey conducted with families. The evaluation team collected and managed the data using REDCap software, and a descriptive analysis was performed to assess the characteristics of the CARA population.

#### Medicaid Claims and CARA Database Linkage

Medicaid records were requested for all infants born in 2020 and 2021 who had a delivery insurance claim, with diagnosis and procedure code information up to six months after birth. The evaluation team determined ICD-10 diagnosis codes that should qualify the infant’s family for CARA support at the time of birth hospitalization to identify records for analysis. (Electronic Supplementary Material 1.)

The Medicaid/CARA linkage was performed in two stages. First, the linkage was performed used a deterministic approach by Medicaid ID number using SAS software, and then, for CARA records not associated with Medicaid ID numbers, the infant’s first name, last name, and date of birth were linked using Match*Pro. The goal of the linkage was to assess whether New Mexico birthing facilities were reporting plans of safe care for infants with documented substance exposure during the birth hospitalization.

#### Program Reports

Monthly program reports were collected via meeting notes and emails from monthly CARA workgroup meetings and evaluation meetings that included representatives from the New Mexico Children, Youth And Families Department, New Mexico Department of Health, Medicaid, and CARA staff responsible for implementing the CARA program. At these meetings participants discussed the policies and procedures of CARA.

#### Family Follow-Up Survey

A follow-up survey of families with a plan of safe care was conducted to assess CARA implementation. Survey questions related to the families’ experience with creating the plan of safe care, implementing the plan, and stigma experienced by pregnant people with substance use disorder. Subject matter experts designed and reviewed the structured survey to assess the validity and suitability of questions. Interns and contractors conducted the survey with the New Mexico Department of Health. The survey was piloted with respondents and modified based on surveyor feedback; pilot responses were excluded from the analysis. The survey was conducted with families who (1) had a child born with a plan of safe care between April and December 2020, (2) could be reached within three call attempts, and (3) were willing to complete the survey. Out of 905 families who received a plan of safe care in that time-period, 143 families completed a survey. Surveys were conducted between 9 months and 1 year after birth. These findings informed which benefits infants exposed to substances in-utero and their families received because of their participation in the CARA program.

*Statement of Ethical Research* This research was conducted in accord with prevailing ethical principles. An Institutional Review Board was not required because the methodology is a public health program evaluation.

## Assessment

### CARA Population

Analysis of the plans of safe care database revealed there were 1,137 plans of safe care in 2020 and 1215 in 2021, a total of 2352. Nearly half of all families with plans of safe care lived in the Metro region (45%), followed by the Southwest (22%), the Northeast (13%), the Northwest (9%), and the Southeast (8%). (See Table 2.) The Northeast region (8.2%) had the highest proportion of all New Mexico births with plans of safe care in 2020, followed by the Southwest region (6.8%), the Northwest (6.0%), the Metro region (5.2%), and the Southeast region with the lowest proportion of 2.5%.

**Table 2:** New Mexico CARA family demographics, New Mexico, 2020

	Count of CARA families, NM, 2020–2021	Proportion of CARA families, NM, 2020–2021 (%)
Region of state		
Metro	1053	45
Southwest	511	22
Northeast	316	13
Northwest	219	9
Southeast	191	8
Unknown	51	2
MCO/New Mexico medicaid insurance provider		
Blue cross blue shield	735	31
Presbyterian	1164	49
Western sky	224	10
Medicaid	144	6
Private	34	1
Uninsured	9	0
Unknown	42	2
Care coordination status		
Received care coordination	881	62.5
Substance exposure classification		
Alcohol, opioids, methamphetamines, and/or cocaine	1074	46
Marijuana only	941	40
Individual substance exposures^a^		
Alcohol	126	5
Benzodiazepines	78	3
Buprenorphine	249	11
Marijuana	1466	62
Methadone	273	12
Methamphetamine	787	33
Nicotine	262	11
Opioids	443	19
Cocaine	123	5
Number of substance exposures		
1	1433	61
2	530	23
3	255	11
4 or more	116	5
Unknown	18	1
Housing at discharge		
Parental home	2010	85
Designated caregivers (e.g., grandparent or aunt)	149	6
Foster home	91	4
Precarious housing (e.g., homeless or unstable housing)	42	2
Facility or shelter	25	1
Unknown	26	1
Other	9	0
New Mexico Children, Youth and Families Department History^b^		
Any family New Mexico Children, Youth and Family Department History	1157	49
Substantiated investigations for families with New Mexico Children, Youth and Families Department History^c^		
One or more substantiated cases	708	30
No substantiated cases	1644	70

^a^Proportions do not total 100% due to some plans of safe care reporting multiple exposures

^b^New Mexico Children, Youth and Family Department history refers to any past investigations related to the mother or father not including the child. These could also include investigations that involved the mother or father when they were a child

^c^Substantiated cases show that the mother or father had at least one prior substantiated case involving them

As Table 2 shows, nearly all families with plans of safe care (96%) had Medicaid– 49% of all families had Presbyterian, followed by 31% with Blue Cross Blue Shield, and 10% with Western Sky, 6% had Medicaid with no listed MCO, 2% had an unknown insurance status, less than 1% of families had private insurance, and less than 1% were uninsured. In comparison, 55% of all New Mexico births had Medicaid. Sixty-three percent of families with a plan of safe care received care coordination; of families not receiving care coordination, 44% showed marijuana as their only exposure.

The substances mandated for reporting and the proportion of plans of safe care reporting those substances include alcohol (5%), benzodiazepines (3%), buprenorphine (11%), marijuana (62%), methadone (12%), methamphetamine (33%), nicotine (11%), opioids (19%), and cocaine (5%). Forty-six percent of plans of safe care involved alcohol, opioids, methamphetamines, or cocaine. While nearly two-thirds of all infants were exposed to marijuana during pregnancy (62%), 40% of infants had marijuana as their only substance exposure. Over half (61%) of all infants with a plan of safe care were exposed to one substance, 23% were exposed to two, 11% were exposed to three, and 5% were exposed to four or more substances.

### Medicaid-CARA Data Linkage

There were 28,604 New Mexico resident infants with a New Mexico Medicaid birth claim born in 2020 and 2021. Of those, 2299 were born in a New Mexico hospital with a diagnosis coded from the birth hospitalization that would qualify them for CARA services, and 1372 (59.7%) had a plan of safe care reported to the state (Table 3).

**Table 3:** Number and proportion of infants born in NM with a Medicaid birth claim that have a CARA-qualifying diagnosis, by region, 2020–2021

Hospital region	Infants with a plan of safe care	Infants with a CARA-qualifying diagnosis at the birth hospitalization	Proportion of eligible infants with a plan of safe care (%)	Total NM occurrent, resident births	2020 NM occurrent, resident births	2021 NM occurrent, resident births	No. of birthing facilities	Medicaid occurrent, resident 2020–2021 births
Metro	870	1351	64.4	17,473	8744	8729	5	10,186
Northeast	146	270	54.1	4600	2301	2299	7	1940
Northwest	80	164	48.8	4240	2249	1991	3	1980
Southeast	69	175	39.4	6222	3249	2973	7	3488
Southwest	207	339	61.1	6928	3544	3384	6	4058
Overall	1372	2299	59.7	39464	20088	19,376	28	21,652^a^

^a^The NM occurrent, resident birth denominator is from the Vital Records database, this differs from the denominator calculated directly from Medicaid claims

Families not covered by Medicaid were excluded from the analysis. The proportion of exposed infants with a plan of safe care varied widely by hospital (between 0% and 76.7%) and by month and year of birth (39.2% to 73.0%), with the lowest proportion occurring the first month of required reporting (January 2020). In 2020, 57.9% and in 2021, 61.4% of eligible families were reported to the CARA program.

### Program Reports on Implementation

Currently, CARA funding is limited to federal funds for New Mexico Children, Youth and Families Department to facilitate data collection for federal reporting requirements. Funding dedicated to support the program’s needs, which includes multiple full-time positions to deliver program management, case management, training, and reporting system development is not currently available. Also, some program resources have been directed toward data requests and media responses related to ideological disagreements with CARA principles. These disagreements are centered primarily on beliefs that prenatal drug exposure should be considered child abuse.

Screening for maternal substance use is not universal or standardized in New Mexico; this has led to prenatal care providers conducting selective screening of pregnant people. Furthermore, estimates of substance exposure during pregnancy in New Mexico are much higher than what is reported. (PRAMS, 2020). Developing plans of safe care also is a comprehensive and time-consuming process, and this has been reported as a barrier to completing the plans of safe care and communicating the plan to families.

New Mexico is a primarily rural state with more than half of the population residing in rural areas. Most of these areas do not have medication-assisted treatment inpatient facilities that accept pregnant people or people with newborns. Only four inpatient programs exist in the state for pregnant and postpartum people who also accept the infant with the postpartum person and only one is in the southern part of the state. Program feedback has indicated that the shortage of programs can result in a lack of screening for substance use disorders during pregnancy in these regions because providers want to avoid identifying substance use in pregnant people if they cannot provide them with appropriate supports.

### Family Follow-Up Survey

Families with a plan of safe care were surveyed to understand their awareness and experience with their plans of care, as well as any bias they may have encountered. Of those surveyed (n = 143), just over half (54%) said that someone talked to them about their plan of safe care and 26% of families surveyed were involved in the making of their plan of safe care. (See Table 4.) Several (n = 8) participants mentioned that they felt “overwhelmed” when they were offered all of these services or were “in a rush to get home” after delivering. Approximately two-thirds of families (65%) received a copy of their plan of safe care, and three-quarters (79%) of families received a call from their insurance provider about care coordination.

**Table 4:** Survey results, New Mexico, 2020

	Count of CARA families, NM, 2020	Proportion of CARA families, NM, 2020 (%)
Did anyone talk to you about your plan of care, which is the plan made when your baby was born to help you with services? (n = 143)		
Yes	77	53.8
No	47	32.9
Don’t know what that is	19	13.3
Were you involved in the making of your plan of care? (n = 137)		
Yes	36	26.3
No	93	67.9
Don’t know what that is	8	5.8
Did you receive a copy of your plan of care? (n = 130)		
Yes	84	64.6
No	35	26.9
Don’t know what that is	11	8.5
Did you receive a call from your insurance provider about care coordination, which is where someone helps you with accessing services, for you and your newborn? (n = 138)		
Yes	109	79
No	29	21
Please tell me if you strongly agree, agree, somewhat agree, disagree, strongly disagree with the following statement: the plan of Care has been useful to you and your family. (n = 130)		
Strongly agree or agree	35	26.9
Somewhat agree	48	36.9
Strongly disagree or disagree	47	36.2
Please tell me if you strongly agree, agree, uncertain, disagree, strongly disagree with the following statement: my health-care team made me feel judged for having used tobacco, alcohol, or drugs during pregnancy. (n = 124)		
Strongly agree or agree	48	38.7
Uncertain	17	13.7
Strongly disagree or disagree	59	47.6
Please tell me if you strongly agree, agree, uncertain, disagree, strongly disagree with the following statement: did you ever hesitate or decide not to access health-care or other services because you were worried about what would happen if providers knew you may have been using tobacco, marijuana, alcohol, or other substances during pregnancy? (n = 116)		
Strongly agree or agree	16	13.8
Uncertain	10	9
Strongly disagree or disagree	90	77.6

When asked if the plan of safe care has been useful to them and their families, 27% of participants agreed or strongly agreed, 37% were uncertain, and 36% disagreed or strongly disagreed.

Thirty-nine percent strongly agreed or agreed that their health-care team made them feel judged for having used tobacco, alcohol, or drugs during pregnancy. Additionally, an estimated 14% of respondents ever hesitated to access healthcare or other services because they were worried about what would happen if providers knew they may have used substances during pregnancy.

## Discussion

In summary, evaluation results assessing plans of safe care reporting, program limitations, and patient experiences have provided key information about how to successfully implement a supportive, nonpunitive policy on perinatal substance use. Providers and birthing facilities are not reporting infants exposed to substances in-utero consistently. The evidence is that 40% of newborns with Medicaid and an ICD-10 code for substance exposure do not have a plan of safe care, although a limitation to using ICD-10 codes to estimate substance exposure is that codes can be entered errantly, or not entered at all. Regardless, the CARA program has not provided enough guidance on how to use reportable ICD-10 diagnoses and this may have resulted in incomplete reporting from hospitals. Increasing the level of training for providers and facilities on plans of safe care reporting based on ICD-10 diagnoses could help birthing facilities create a standardized process, as well as reduce bias by removing the current practice of selective reporting by hospital staff. Furthermore, the inability to provide treatment due to the lack of medication-assisted treatment facilities for pregnant and postpartum people may have also resulted in reduced reporting. In addition, when plans of safe care were created, hospital staff were not communicating the plan of safe care process consistently to families with substance-exposed newborns. Hospital staff need more time to complete the plan of safe care in addition to the other discharge documents they prepare after birth hospitalizations.

Currently, one birthing facility in New Mexico has implemented universal substance screening. Two measures can ease this process. Prenatal screening for substance exposure through universal screening policies via a validated verbal screening questionnaire (e.g., The 5Ps Prenatal Substance Abuse Screen for Alcohol and Drugs) could help identify families, standardize reporting and reduce bias in screening, and increase the number of referrals to prenatal services to engage families earlier on than currently is the case. In addition, the CARA program needs to improve their guidance on specific concerns that must be reported to New Mexico Children, Youth and Families Department for an abuse/neglect assessment so that hospital staff are positioned to reduce the number of families they report who have an infant born with a substance exposure but no other safety concerns. Trainers need to improve guidance to health-care practitioners and social workers to also ensure that infants living in unsafe circumstances are reported to New Mexico Children, Youth and Families Department for an assessment.

Families continued to experience bias from health-care providers and hesitated to access care while pregnant and using substances, despite CARA policy. This underscores the need for more ongoing work to reduce stigma in hospital settings regarding perinatal substance use.

Another key limitation to implementing the CARA policy was limited funding because CARA requires a more robust programmatic structure including more positions and a program plan to support compliance with the CARA statute. Appropriate supports are a must to facilitate implementation of the CARA policy among the large partnering organizations—Medicaid, New Mexico Children, Youth and Families Department, New Mexico Department of Health, and all birthing facilities. To implement CARA effectively, more full-time staff positions are needed to: (1) manage the program; (2) facilitate interorganizational collaboration among partners carrying out the policy; (3) conduct case management for CARA families (in addition to MCO care coordination); (4) oversee development of reporting systems (including the ability to track service referrals and evaluate whether families access those services); and 5) train staff in hospitals and MCOs.

## Conclusion

The implementation of CARA in New Mexico has provided care coordination to most families who received a plan of safe care. However, the lack of funding, shortages of critical full-time staff, and programmatic structure has limited the capacity of this program to reach all substance-exposed newborns in the state. The state urgently needs improved guidelines that outline which ICD-10 diagnoses codes require healthcare providers to create a plan of safe care and report it. When this happens, the reporting process will improve and biased screening will be reduced. Ideally, the plan of safe care process would occur during the prenatal period via a verbal screening tool conducted with every pregnant person. Increased healthcare provider training also should be conducted to reduce bias related to perinatal substance use. Further assessments of hospital staff needs and constraints in creating plans of safe care and subsequent process changes may also improve reporting. These changes would improve the efficacy of the New Mexico CARA program and ultimately ensure that families with substance-exposed newborns receive the nonpunitive, equitable care they deserve.

### Supplementary Information

Below is the link to the electronic supplementary material.

Supplementary file1 (PDF 34 KB)

## Data Availability

Not applicable.

## Footnotes

1 All subsequent references to CARA that do not explicitly refer to federal CARA legislation refer to New Mexico CARA.

## Code Availability

SAS code used for data linkage available upon request.
