# Supplementary material for: An Implementation Evaluation of the Comprehensive Addiction Recovery Act (CARA) Policy in New Mexico
Source: Matern Child Health J. 2023 Oct 18;27(Suppl 1):113–21. doi: 10.1007/s10995-023-03787-1 (PMC10691990; doi:10.1007/s10995-023-03787-1)
Supplement: Supplementary file 1 — Supplementary file1 (PDF 34 KB) [file 10995_2023_3787_MOESM1_ESM.pdf]

Article Title: An Implementation Evaluation of the Comprehensive Addiction Recovery Act (CARA) Policy in New Mexico
Journal Name: Maternal and Child Health Journal
Author Names: Nicholas Sharp and Jessica Fuchs
Affiliation: New Mexico Department of Health
E-mail address of the corresponding author: Nicholas.Sharp@doh.nm.gov and Jessica.Fuchs@doh.nm.gov

I. ICD-10 Diagnosis Codes identifying newborns affected by substance exposure

**ICD-10 Diagnosis Code**	**Code description**
P04.14	Newborn affected by maternal use of opiates
P04.16	Newborn affected by maternal use of amphetamines
P04.17	Newborn affected by maternal use of sedative-hypnotics
P04.1A	Newborn affected by maternal use of anxiolytics
P04.3	Newborn affected by maternal use of alcohol
P04.40	Newborn affected by maternal use of unspecified drugs of addiction
P04.41	Newborn affected by maternal use of cocaine
P04.42	Newborn affected by maternal use of hallucinogens
P04.49	Newborn affected by maternal use of other drugs of addiction
P04.81	Newborn affected by maternal use of cannabis
P96.1	Neonatal withdrawal symptoms from maternal use of drugs of addiction
P96.2	Withdrawal symptoms from therapeutic use of drugs in newborn
Q86.0	Fetal alcohol syndrome
